# Effects of AST‐120 on muscle health and quality of life in chronic kidney disease patients: results of RECOVERY study

**DOI:** 10.1002/jcsm.12874

**Published:** 2021-12-03

**Authors:** Ran‐hui Cha, Seok Hui Kang, Mi Yeun Han, Won Suk An, Su‐Hyun Kim, Jun Chul Kim

**Affiliations:** ^1^ Department of Internal Medicine National Medical Center Seoul Republic of Korea; ^2^ Department of Internal Medicine Yeungnam University College of Medicine Daegu Republic of Korea; ^3^ Department of Internal Medicine Hallym University Hangang Sacred Heart Hospital Seoul Republic of Korea; ^4^ Department of Internal Medicine Dong‐A University College of Medicine Busan Republic of Korea; ^5^ Department of Internal Medicine Chung‐Ang University Hospital, Chung‐Ang University College of Medicine Seoul Republic of Korea; ^6^ Department of Internal Medicine, CHA Gumi Medical Center CHA University Gumi Republic of Korea

**Keywords:** AST‐120, Chronic kidney disease, Gait speed, Handgrip strength, Quality of life, Sarcopenia

## Abstract

**Background:**

The prevalence of sarcopenia is increased with declining renal function. Elevated serum indoxyl sulfate levels are associated with poor skeletal muscle conditions. We aimed to determine the effects of AST‐120, the oral adsorbent of indoxyl sulfate, on sarcopenia and sarcopenia‐associated factors in chronic kidney disease patients.

**Methods:**

This was a 48 week, randomized controlled, parallel group, open‐label, multicentre trial (*n* = 150). The participants were randomly assigned in a 1:1 ratio to the control (CON) and AST‐120 (Renamezin®, REN) groups. Outcome measurements were performed at baseline and every 24 weeks for 48 weeks. The primary outcome was gait speed difference ≥0.1 m/s between the two groups, and secondary outcomes included hand grip strength, muscle mass, and health‐related quality of life.

**Results:**

A difference of gait speed ≥0.1 m/s was not observed during the study period. The mean dynamic‐start gait speed in the REN group increased from baseline to 48 weeks (1.04 ± 0.31 to 1.08 ± 0.32 m/s, *P* = 0.019). The static‐start gait speed changed by −0.024 and 0.04 m/s (*P* = 0.049) in the CON and REN groups over 48 weeks, respectively. Hand grip strength decreased during the first 24 weeks and did not significantly change over the next 24 weeks in either group. The proportion of low muscle mass or sarcopenia at baseline was larger in the REN group than in the CON group, but the difference attenuated over the study period [low muscle mass and sarcopenia in the CON and REN groups at baseline, 4.0% vs. 18.9% (*P* = 0.004) and 2.7% vs. 13.5% (*P* = 0.017); at 24 weeks, 2.9% vs. 13.6% (*P* = 0.021) and 1.4% vs. 10.5% (*P* = 0.029); and at 48 weeks, 7.6% vs. 12.9% (*P* = 0.319) and 4.5% vs. 8.1% (*P* = 0.482), respectively]. Bodily pain, vitality, symptoms/problems, and cognitive function in the REN group improved, while the quality of social interactions and the kidney disease effects in the CON group aggravated from baseline to 48 weeks. Interaction between time and group was evident only in symptoms/problems, cognitive function, and kidney disease effects.

**Conclusions:**

The addition of AST‐120 to standard treatment in chronic kidney disease patients did not make a significant difference in gait speed, although AST‐120 modestly had beneficial effects on gait speed change and quality of life and showed the potential to improve sarcopenia. (clinicaltrials.gov: NCT03788252).

## Introduction

Sarcopenia is characterized by a loss of muscle mass and strength, a decreased quality of life (QoL) with age, morbidities, and immobility and is associated with protein‐energy wasting in chronic kidney disease (CKD) and end‐stage kidney disease patients.^1^ The prevalence of sarcopenia is increased with declining kidney function.^1^, ^2^ CKD is associated with chronic low‐grade inflammation leading to progressive weight loss, muscle weakness, and the loss of the ability to exercise. Inflammatory cytokines, oxidative stress, and the inactivity‐mediated destruction of protein homeostasis result in the catabolic destruction of structural and functional proteins, skeletal muscle wasting, and a decrease in exercise capacity.^3^, ^4^ Mitochondrial dysfunction of skeletal muscles in CKD is also considered a cause of the loss of muscle mass and exercise capacity.^5^, ^6^, ^7^

Uraemic toxins enter target cells via specific transporters, such as organic anion transporters (OATs).^8^, ^9^, ^10^ IS also enters various cells via OATs (OAT1 and OAT3), and OATs are also expressed in muscles.^11^, ^12^, ^13^ Protein‐bound uraemic toxins, including IS and p‐cresyl sulfate, exert their toxicity via the activation of cellular NAD(P)H oxidase, which results in the overproduction of reactive oxygen species (ROS) and inflammatory cytokines.^14^, ^15^ IS accumulation in muscle cells and subsequent superoxide production and the up‐regulation of inflammatory cytokines such as tumour necrosis factor (TNF)‐α, interleukin (IL)‐6, and transforming growth factor‐β induce muscle wasting through myostatin and atrogin‐1.^13^ And these changes are associated with mitochondrial dysfunction, which is mediated by metabolic alterations, that is, the up‐regulation of antioxidative responses and down‐regulation of energy‐generation pathways.^16^

A significant inverse association between plasma IS levels and skeletal muscle mass has been found in CKD patients.^16^ In addition, IS down‐regulates Klotho expression through ROS‐associated nuclear factor‐kB activation in the kidney.^17^, ^18^ Klotho levels and skeletal muscle physiology are closely related.^19^, ^20^

AST‐120 reduces the accumulation of IS in organs, including skeletal muscles^21^; increases Klotho expression; and inhibits cell senescence in uraemic mouse and rat.^22^ AST‐120 in *in vivo* and *in vitro* experiments also prevented the CKD‐induced physical inactivity mainly by maintaining mitochondrial function, suppressing atrogin‐1/myostatin expression, and recovering Akt phosphorylation in skeletal muscles.^23^

However, the effect of AST‐120 on sarcopenia in CKD patients has never been studied. Therefore, we aimed to determine the effects of AST‐120 on sarcopenia and sarcopenia‐associated factors in CKD patients.

## Materials and methods

### Study design

This was a 48 week, randomized controlled, parallel group, open‐label, multicentre trial. This study was registered in clinicaltrials.gov on 27 December, 2018 [RolE of AST‐120 in sarCOpenia preVEntion in pRe‐dialYsis chronic kidney disease patients (RECOVERY): NCT03788252]. The participants were randomly assigned in a 1:1 ratio to the control (CON) and AST‐120 (Renamezin®, REN) groups. Measurements were taken at baseline and every 24 weeks for 48 weeks; the measurements included vital sign recordings, blood and urine laboratory examination findings, body composition including bioelectric impedance study findings, physical performances, and questionnaire responses. This study was approved by the institutional review boards of the participating hospitals. We conducted this study in compliance with the principles of the Declaration of Helsinki. All participants provided informed consents.

### Study outcomes

The primary outcome was physical performance (gait speed difference ≥0.1 m/s between the two groups) in CKD patients. We chose the gait speed as the primary outcome because of following reasons^24^: (1) it is hard to reverse muscle mass due to old age and advanced kidney dysfunction of participants; (2) gait speed serves as a core indicator of health and function in ageing and diseases; (3) gait speed is a quick and reliable measure of functional capacity with high interrator and test–retest reliability. The secondary outcomes included muscle mass and strength, physical activity level, levels of inflammatory and muscle‐related markers (IS, TNF‐α, IL‐6, and myostatin), health‐related QoL, and renal function [serum creatinine (SCr) and estimated glomerular filtration rate (eGFR)].

### Recruitment and population

A total of 150 CKD patients were recruited from six general hospitals in Korea.

Patients were eligible for inclusion in this study only if the patient (1) had pre‐dialysis CKD and was aged 20 years or older; (2) had stable renal function with an SCr level between 2.0 and 5.0 mg/dL or MDRD (or CKD‐EPI) eGFR between 15 and 60 mL/min/1.73 m^2^ for 3 months; (3) had a serum albumin level higher than 3.0 g/dL; (4) was naïve to AST‐120 during the 4 weeks before screening; (5) did not have significant changes in medical treatment within the 4 weeks before screening; (6) could ambulate without any help from caregivers except for using auxiliary devices; and (7) could thoroughly understand the protocol and sign the informed consent form.

Patients were excluded for any of the following reasons: (1) they had passage disorders in the gastrointestinal tract and uncontrolled constipation; (2) they were kidney transplantation recipients or were expected to undergo dialysis or kidney transplantation within 3 months after enrolment; (3) they were taking immune‐suppressive agents; (4) they had active ulcers or oesophageal varices; (5) they had an uncontrolled blood pressure of ≥180/110 mmHg; (6) they had acute or subacute cardiovascular diseases within the last 3 months; (7) they had active infections or uncontrolled inflammatory diseases; (8) they had abnormal aspartate‐ and alanine‐ aminotransferase >2.5 times the upper normal limit; (9) they had an uncontrolled blood sugar level (fasting glucose >250 mg/dL or HbA1c > 10.0%); (10) they had a progressive malignancy; (11) they were pregnant, lactating, or planning to be pregnant during the study period; (12) they had severe retinopathies such as proliferative diabetic retinopathy or vitreal haemorrhage; (13) they had orthopaedic disorders that can be aggravated by physical activity; (14) they underwent leg amputation but did not wear a prosthetic legs or they showed claudication; (15) they were participating in other clinical studies; and (16) they were considered ineligible for the study by the investigators.

### Randomization and interventions

The patients who were enrolled were randomly assigned at a ratio of 1:1 to the CON and REN groups. We performed the block randomization (6 blocks of size 25 for 1:1 allocation). An independent statistician used SAS (version 9.4) to generate random numbers, and only an independent physician had the pregenerated codes. All the participants received standard care, including angiotensin‐converting enzyme inhibitors and/or angiotensin receptor blockers and lipid modifiers. The participants in the REN group were instructed to self‐administer the drug orally in three divided doses (Renamezin® 21 capsules a day) for a total of 6 g a day. Designated researchers counted the residual capsules of AST‐120 at every visit to assess compliance.

### Sample size

The sample size was estimated such that a mean between‐treatment group difference in gait speed of ≥0.1 m/s could be detected. We assumed a standard deviation (SD) of gait speed of 0.2 m/s. Assuming a two‐tailed hypothesis, an alpha value of 0.05 and a desired power of 80%, 50 participants were found to be needed per group to complete the study. Allowing for a 34% dropout rate, we calculated the required sample size to be 75 patients per treatment group.

### Measurements

#### Six‐metre gait speed test

The participants walked at their usual walking speed after the examiner instructed to do so. The participants could use walking aids such as walking sticks, canes, or walkers. We adopted both static and dynamic start methods. For the static start method, the participants stood on a 6 m start line and started walking after examiner instructed to do so. For the dynamic start method, the participants started walking 2 m prior to the measurement point (acceleration zone) and ended 2 m after the measurement point (deceleration zone). The participants repeated each type of walking speed test two times, and all data including the mean were analysed.

#### Hand grip strength

Hand grip strength (HGS) was assessed by using digital hand dynamometers (T.K.K.5401, Takei Scientific Instruments Co. Ltd., Niigata, Japan) in both hands. The participants measured HGS in two positions: sitting and standing. The participants stood upright with the shoulders facing forward without rotation, the elbows extended, and the wrists in neutral flexion for the standing position measurements. The participants were seated, with the shoulders facing forward without rotation, the elbows flexed to 90°, and the wrists in neutral flexion for the sitting position HGS measurements. The participants were encouraged to grasp the device strongly three times at intervals of 30 s in each position and each hand. The largest HGS value for each method was used in the analysis.

#### Body composition

The participants were fasted at least 8 h before the body composition test but could take essential medications with a small amount of water (<100 mL). Body composition was measured by using an InBody S10 (Seoul, South Korea), an instrument based on bioelectrical impedance analysis.

#### Laboratory tests

Parameters including IS, TNF‐α, IL‐6, myostatin, intact parathyroid hormone (iPTH), and 25‐OH‐vitamin D levels in serum were measured at the central laboratory institution (Seoul Clinical Laboratories, Yongin‐si, Gyeonggi‐do, Korea). The TNF‐α, IL‐6, and myostatin levels were assessed by an enzyme‐linked immunosorbent assay (ELISA) using HSTA00D (Human TNF‐α Quantikine HS ELISA), HS600C (Human IL‐6 Quantikine HS ELISA Kit), and DGDF80 (GDF‐8/Myostatin Quantikine ELISA Kit, R&D Systems). The levels of iPTH and 25‐OH‐vitamin D were measured by electrochemiluminescence immunoassay and chemiluminescence immunoassay using a Cobas E801 analyser (Roche Diagnostics GmbH, Mannheim, Germany). The serum total IS levels were measured using a high‐performance liquid chromatography‐fluorescence detector (HPLC‐FLD, Agilent 1260 series; Agilent Technologies, Santa Clara, CA, USA). Other blood and urine data were measured at each research institution.

#### Questionnaire survey

We used the Charlson comorbidity index to assess the comorbidity status of the participants. We used version 1.3 of the kidney disease QoL (KDQOL) short form to evaluate health‐related QoL. A short, self‐administered version of the international physical activity questionnaire (IPAQ) was used to assess the physical activity level of the individual over the past 7 days.

#### Statistical methods

We performed the primary analysis by intention‐to‐treat (ITT; including data on the patients who were randomly assigned to a group and underwent any study outcome evaluations) and per‐protocol (PP; including data on the patients who were randomly assigned to a group and completed all the data collections without major protocol deviations) methods. We conducted all statistical analyses by using PASW advanced statistics (SPSS Inc, Chicago, IL, USA) version 20.0. The data are reported as the mean, standard deviation, or percentage frequency.

We used Student's *t*‐test (or Mann–Whitney *U* tests) and paired *t* tests (or Wilcoxon signed‐rank tests) for the continuous variables depending on whether the data were normally distributed, and the *χ* ^2^chi‐square test for the categorical variables. The between‐group differences in the outcome measures after the intervention were assessed by using repeated measure analysis of variance. Multiple regression analysis was conducted to determine the statistical significance of each variable with respect to the outcomes. The Spearman rank test or Pearson product–moment correlation coefficient analysis was used to analyse the associations between the clinical data and outcome measures. We considered *P* < 0.05 (two sided) statistically significant.

## Results

### Study population and baseline characteristics

**Figure 1:**
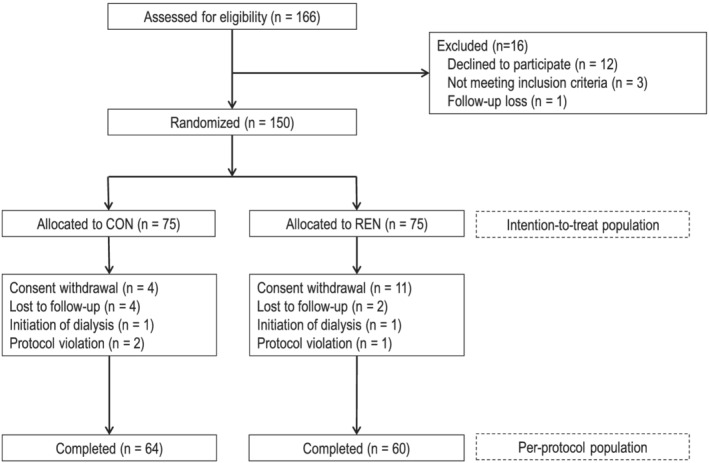
Flow chart of study participant enrolment, randomization, and analysis.

The first participant was enrolled on November 11, 2018, and the last follow‐up was performed on June 16, 2020. A total of 150 patients were randomly assigned to the CON and REN groups and underwent any study outcome evaluations (ITT population). In total, 124 patients completed the follow‐up and all the study outcomes (PP population) (*Figure* 1). The mean AST‐120 compliance rates of the ITT and PP populations were 85.9% and 87.3%, respectively. The baseline characteristics were not significantly different between the two treatment groups in the ITT analysis (*Table* 1) and PP analysis.

**Table 1:** Baseline characteristics of intention‐to‐treat population

	Total (*n* = 150)	CON (*n* = 75)	REN (*n* = 75)	*P* value^a^
Age (years)	65.0 ± 10.8	65.9 ± 10.7	64.1 ± 10.8	0.318
Sex (men)	97 (64.7%)	52 (69.3%)	45 (60.0%)	0.232
Diabetes mellitus	76 (50.7%)	36 (48.0%)	40 (53.3%)	0.514
Modified CCI score	3.9 ± 1.9	3.8 ± 1.8	3.9 ± 2.0	0.667
Haemoglobin (g/dL)	12.3 ± 2.1	12.3 ± 2.0	12.4 ± 2.1	0.826
Albumin (g/dL)	4.3 ± 0.4	4.3 ± 0.4	4.3 ± 0.3	0.242
Calcium (mg/dL)	9.1 ± 0.6	9.1 ± 0.6	9.1 ± 0.5	0.707
Phosphorus (mg/dL)	3.5 ± 0.6	3.5 ± 0.6	3.5 ± 0.6	0.483
hs‐CRP (mg/dL)	0.5 ± 1.2	0.4 ± 1.0	0.5 ± 1.4	0.599
Blood urea nitrogen (mg/dL)	32.0 ± 11.0	32.3 ± 11.8	31.6 ± 10.3	0.694
Creatinine (mg/dL)	2.1 ± 0.7	2.1 ± 0.7	2.1 ± 0.8	0.668
Total CO_2_ (mmol/L)	23.8 ± 3.5	23.9 ± 3.0	23.7 ± 3.9	0.806
Sodium (mmol/L)	140 ± 2	140 ± 2	140 ± 2	0.860
Potassium (mmol/L)	4.8 ± 0.6	4.9 ± 0.6	4.8 ± 0.6	0.651
Chloride (mmol/L)	106 ± 4	106 ± 4	106 ± 4	0.888
iPTH (ng/mL)	86.5 ± 67.9	88.8 ± 80.6	84.2 ± 52.6	0.683
eGFR (CKD‐EPI)	33.8 ± 12.5	33.0 ± 12.0	34.6 ± 12.9	0.442
Pr/Cr ratio	1.3 ± 1.5	1.5 ± 1.7	1.2 ± 1.2	0.299

Data are expressed as numbers (percentages) for categorical variables and means ± standard deviations for continuous variables. The *P* values were tested with *t* test for continuous variables, and Pearson *χ* ^2^ test or Fisher exact test was used to analyse categorical variables.

Abbreviations: CCI, Charlson comorbidity index; CKD‐EPI, Chronic Kidney Disease‐Epidemiology Collaboration; eGFR, estimated glomerular filtration rate; hs‐CRP, high sensitivity‐C‐reactive protein; iPTH, intact parathyroid hormone; Pr/Cr, spot urine protein to creatinine.

^a^ Between the CON and the REN.

### Indoxyl sulfate level

**Figure 2:**
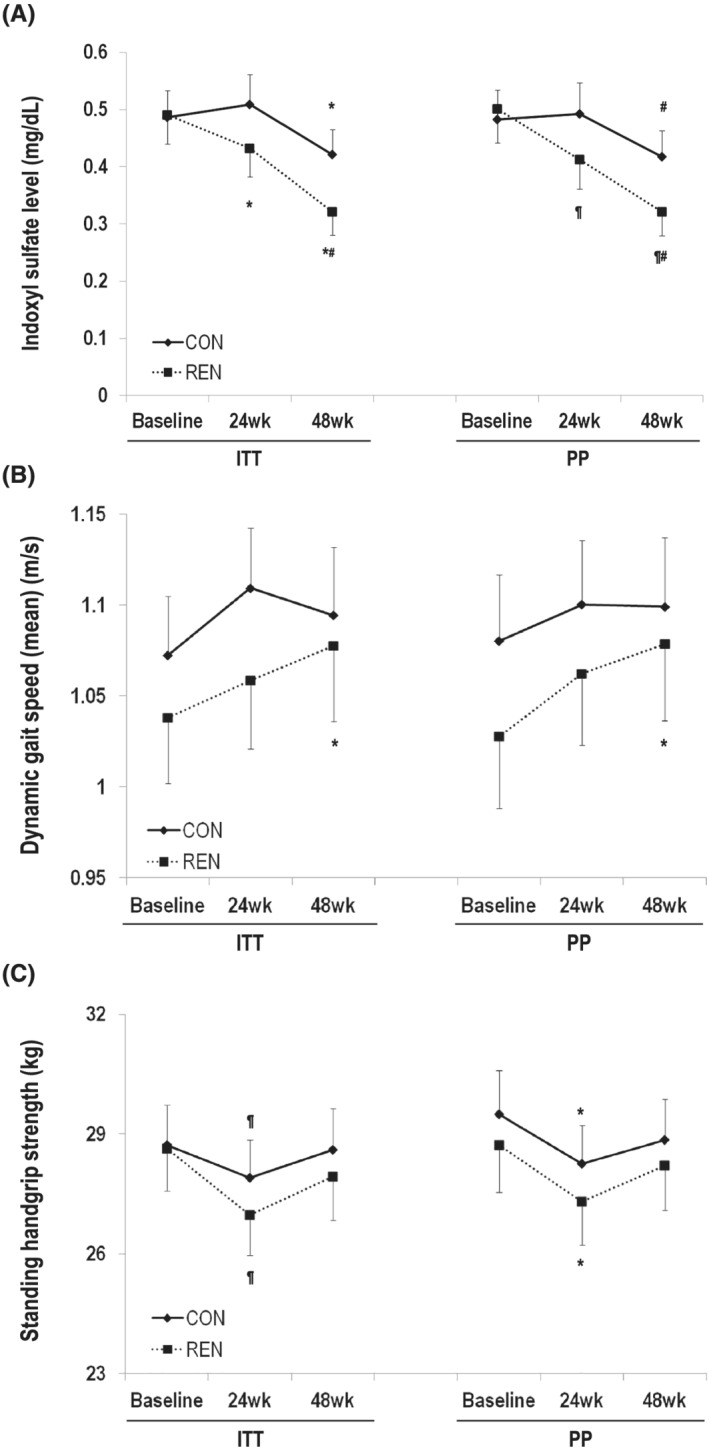
Changes of indoxyl sulfate level (*A*), gait speed (*B*), and standing handgrip strength (*C*) from baseline to 24 and 48 weeks.Data were expressed as mean and standard error. *P < 0.05 vs. baseline, ^¶^ *P* < 0.01 vs. baseline, ^#^ *P* < 0.05 vs. 24 weeks. ITT, intention‐to‐treat; PP, per‐protocol.

In the ITT analysis, the serum IS levels in the CON and REN groups were 0.50 ± 0.42 and 0.50 ± 0.46 mg/dL at baseline, 0.50 ± 0.43 and 0.41 ± 0.40 mg/dL at 24 weeks, and 0.42 ± 0.36 and 0.32 ± 0.32 mg/dL at 48 weeks, respectively (*Figure* 2A). The serum IS level decreased over 48 weeks in both groups, and the interaction of the treatment group with IS level was weak (*P*_time_ < 0.001, *P*_timeXtreat_ = 0.065). However, in the PP analysis, the interaction of the treatment group with IS level was statistically significant (*P*_timeXtreat_ = 0.046).

### Primary outcome—gait speed

The mean dynamic start gait speeds in the CON and REN groups are presented in *Table* 2 and *Figure* 2B. A difference of gait speed ≥0.1 m/s between the two groups was not observed during the study period (1.07 ± 0.28 and 1.04 ± 0.31 m/s at baseline, 1.11 ± 0.28 and 1.06 ± 0.31 m/s at 24 weeks, and 1.10 ± 0.30 and 1.08 ± 0.32 m/s at 48 weeks in the CON and REN groups, respectively). The mean dynamic‐start gait speed in the REN group increased from baseline to 48 weeks (1.04 ± 0.31 to 1.08 ± 0.32 m/s, *P* = 0.019), and PP analysis showed similar statistically significant results (1.03 ± 0.30 to 1.08 ± 0.33 m/s, *P* = 0.018) (*Figure* 2B).

**Table 2:** Comparison of gait speed and handgrip strength between treatment groups

	Baseline			24 weeks			48 weeks		
	CON	REN	*P* value	CON	REN	*P* value	CON	REN	*P* value
ITT population									
Dynamic‐start gait speed (mean, m/s)	1.07 ± 0.28	1.04 ± 0.31	0.484	1.11 ± 0.28	1.06 ± 0.31	0.310	1.10 ± 0.30	1.08 ± 0.32*	0.764
Static‐start gait speed (mean, m/s)	1.05 ± 0.26	1.01 ± 0.22	0.398	1.05 ± 0.21	1.03 ± 0.22	0.494	1.04 ± 0.23	1.04 ± 0.22*	0.896
Dynamic‐start gait speed (faster, m/s)	1.10 ± 0.29	1.06 ± 0.32	0.432	1.13 ± 0.28	1.08 ± 0.32	0.328	1.11 ± 0.31	1.10 ± 0.34*	0.873
Static‐start gait speed (faster, m/s)	1.08 ± 0.28	1.05 ± 0.23	0.432	1.07 ± 0.22	1.05 ± 0.23	0.579	1.06 ± 0.25	1.07 ± 0.24*	0.820
Standing HGS (kg)	28.7 ± 8.75	28.6 ± 9.24	0.959	27.9 ± 7.85	27.0 ± 8.22*	0.495	28.6 ± 8.33	27.9 ± 8.67	0.657
Sitting HGS (kg)	28.6 ± 8.75	28.0 ± 9.07	0.719	27.5 ± 7.70	26.5 ± 8.06*	0.465	28.2 ± 7.93	27.5 ± 9.09	0.648
PP population									
Dynamic‐start gait speed (mean, m/s)	1.08 ± 0.29	1.03 ± 0.30	0.324	1.10 ± 0.28	1.06 ± 0.31	0.475	1.10 ± 0.30	1.08 ± 0.33*	0.717
Static‐start gait speed (mean, m/s)	1.06 ± 0.26	1.00 ± 0.21	0.217	1.05 ± 0.22	1.03 ± 0.22	0.632	1.04 ± 0.23	1.04 ± 0.23*	0.958
Dynamic‐start gait speed (faster, m/s)	1.11 ± 0.30	1.05 ± 0.31	0.301	1.12 ± 0.29	1.08 ± 0.32	0.507	1.12 ± 0.31	1.11 ± 0.34*	0.825
Static‐start gait speed (faster, m/s)	1.09 ± 0.28	1.03 ± 0.22	0.217	1.07 ± 0.22	1.06 ± 0.23	0.710	1.06 ± 0.25	1.07 ± 0.24*	0.889
Standing HGS (kg)	29.5 ± 8.80	28.7 ± 9.17	0.634	28.3 ± 7.69	27.3 ± 8.42*	0.513	28.9 ± 8.14	28.2 ± 8.68	0.668
Sitting HGS (kg)	29.2 ± 8.87	28.3 ± 7.69	0.463	27.7 ± 7.61	26.8 ± 8.23*	0.525	28.4 ± 7.73	27.7 ± 9.11	0.659

Data are expressed as means ± standard deviations. The comparison between groups was tested with *t* test and comparison with baseline value was tested with paired *t* test.

* *P* < 0.05 vs. baseline value.

Abbreviations: HGS, handgrip strength; ITT, intention‐to‐treat; PP, per‐protocol.

AST‐120 also showed a beneficial effect on the change of static‐start gait speed during the study period. The faster static‐start gait speed in the CON and REN groups changed over 48 weeks by −0.024 ± 0.204 and 0.04 ± 0.152 m/s (*P* = 0.049) according to the ITT analysis and −0.025 ± 0.205 and 0.038 ± 0.152 m/s (*P* = 0.058) according to the PP analysis, respectively. Multiple regression analysis showed that AST‐120 tended to increase the static‐start gait speed, although change was marginally statistically significant after adjustments for age, sex, primary disease, and physical activity (95% confidence interval 0.000–0.128, *P* = 0.051 and 95% confidence interval −0.003 to 0.128, *P* = 0.061 for ITT and PP analysis, respectively) (*Table* S1).

### Secondary outcomes

#### Handgrip strength and muscle mass

Hand grip strength at each time point is presented in *Table* 2 and *Figure* 2B. Standing HGS decreased from baseline to 24 weeks in both groups and did not change during the latter 24 weeks (28.7 ± 8.75 and 28.6 ± 9.24 kg at baseline, 27.9 ± 7.85 and 27.0 ± 8.22 kg at 24 weeks, and 28.6 ± 8.33 and 27.9 ± 8.67 kg at 48 weeks in the CON and REN groups, respectively). The interaction of the treatment group with standing HGS was not significant (*P*_timeXtreat_ = 0.875). Standing HGS changed similarly in men and all participants (Supporting *Figure* S1A). In women, there was no significant change in standing HGS over the study period (*Figure* S1B). PP analysis showed similar results. In addition, sitting HGS showed similar results in the two treatment groups and in both sexes.

In the ITT analysis, the skeletal muscle index (SMI) in the CON and REN groups was 7.8 ± 1.2 and 7.6 ± 1.3 kg/m^2^ at baseline, 8.0 ± 1.1 and 7.6 ± 1.3 kg/m^2^ at 24 weeks, and 7.9 ± 1.2 and 7.6 ± 1.3 kg/m^2^ at 48 weeks, respectively. The interaction of the treatment group with SMI was not significant (*P*_timeXtreat_ = 0.616). There was no significant change in SMI from baseline to 48 weeks in either group. PP analysis showed similar results of SMI.

#### Sarcopenic components and sarcopenia

**Figure 3:**
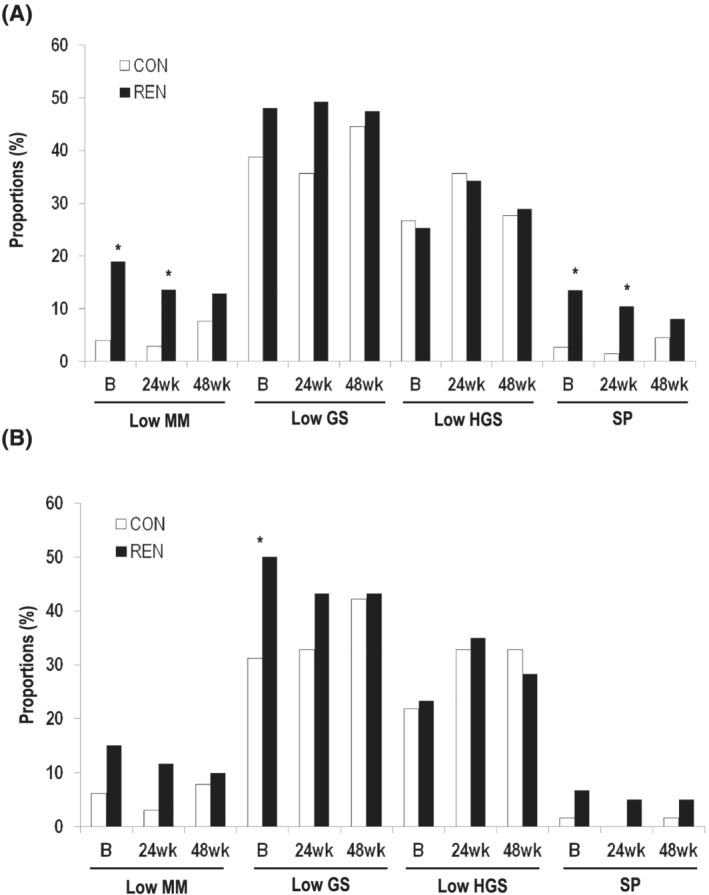
The proportions of low muscle mass (SMI), slow gait speed, weak handgrip strength, and sarcopenia according to 2019 AWGS in the ITT (*a*) and PP (*B*) groups. *P < 0.05. AWGS, Asian working group for sarcopenia; GS, gait speed; HGS, handgrip strength; ITT, intention‐to‐treat; PP, per‐protocol; MM, muscle mass; SP, sarcopenia.

The proportions of patients with low muscle mass (SMI), a slow gait speed (mean dynamic‐start), weak standing HGS, and sarcopenia according to the 2019 Asian Working group for Sarcopenia (AWGS) in the CON and REN groups are presented in *Figure* 3A. The proportion of patients with low muscle mass or sarcopenia at baseline was larger in the REN group than in the CON group, but the difference between the two groups attenuated over the study period (at baseline, 4.0% vs. 18.9% (*P* = 0.004) and 2.7% vs. 13.5% (*P* = 0.017); at 24 weeks, 2.9% vs. 13.6% (*P* = 0.021) and 1.4% vs. 10.5% (*P* = 0.029); and at 48 weeks, 7.6% vs. 12.9% (*P* = 0.319) and 4.5% vs. 8.1% (*P* = 0.482), respectively). The PP and ITT analysis showed similar results (*Figure* 3B).

#### Quality of life scores

There were no significant differences in the QoL scores at baseline between the two groups in the ITT analysis (*Table* 3). The overall health ratings improved from baseline to 24 and 48 weeks in both groups. Bodily pain improved in the REN group from baseline to 48 weeks. According to the kidney disease‐specific scores, the symptoms/problems and cognitive function in the REN group improved from baseline to 48 weeks. The quality of social interactions in the CON group was poorer at 48 weeks than at baseline. The trends for the overall health ratings, bodily pain, symptoms/problems, cognitive function, and quality of social interactions were similar between the ITT and PP analysis (*Table* S2). The vitality in the REN group was improved while the kidney disease effects in the CON group were poorer at 48 weeks than at baseline in the PP analysis. Interaction between time and group was evident only in symptoms/problems, cognitive function, and kidney disease effects, but not in overall health rating, bodily pain, vitality, and quality of social interaction in both the ITT and PP analyses (*Tables* 3 and S2).

**Table 3:** Comparison of quality of life scale scores between treatment groups in intention‐to‐treat population

Short form‐6 scale	Baseline			6 M			12 M			*P* for interaction		
	CON	REN	*P*	CON	REN	*P*	CON	REN	*P*	Time	Group	Time–group
PF	80.4 ± 18.9	76.0 ± 23.7	0.250	78.8 ± 20.3	78.0 ± 20.4	0.805	78.5 ± 25.2	79.8 ± 18.7	0.746	0.786	0.695	0.158
RP	75.4 ± 33.5	68.0 ± 39.8	0.263	77.7 ± 35.9	65.2 ± 2.6	0.076	71.2 ± 41.7	68.0 ± 40.6	0.672	0.782	0.202	0.290
BP	78.0 ± 22.6	75.2 ± 24.4	0.506	81.5 ± 23.2	77.5 ± 24.6	0.349	78.8 ± 25.0	82.6 ± 21.0*	0.357	0.112	0.773	0.162
GH	44.3 ± 17.0	43.4 ± 16.0	0.769	45.5 ± 16.9	46.8 ± 18.5	0.671	44.3 ± 16.5	45.2 ± 18.2	0.762	0.197	0.862	0.644
VT	51.4 ± 18.2	48.4 ± 17.6	0.359	49.8 ± 16.0	51.1 ± 18.8	0.677	51.5 ± 16.2	51.8 ± 16.0	0.927	0.431	0.861	0.260
SF	87.9 ± 21.5	82.8 ± 23.3	0.204	84.6 ± 21.2	82.2 ± 22.4	0.530	82.7 ± 23.2	83.0 ± 23.1	0.942	0.425	0.472	0.413
RE	77.4 ± 38.7	67.8 ± 43.0	0.186	74.4 ± 41.2	69.4 ± 43.2	0.510	71.3 ± 42.0	63.9 ± 45.3	0.347	0.323	0.259	0.754
MH	65.2 ± 15.3	61.5 ± 15.5	0.185	65.5 ± 14.4	61.8 ± 15.1	0.169	65.4 ± 14.2	60.6 ± 15.7	0.076	0.885	0.069	0.902
OHR	26.9 ± 16.7	25.0 ± 17.1	0.524	31.5 ± 19.9*	34.0 ± 19.4*	0.481	33.1 ± 20.8*	33.6 ± 20.9*	0.887	<0.001	0.899	0.402
PCS	69.5 ± 18.4	65.7 ± 20.8	0.272	70.9 ± 19.8	66.9 ± 20.7	0.268	68.2 ± 22.8	68.9 ± 19.3	0.847	0.644	0.461	0.181
MCS	70.5 ± 18.3	65.1 ± 19.9	0.119	68.6 ± 18.5	66.1 ± 20.6	0.485	67.7 ± 18.9	64.8 ± 20.4	0.411	0.588	0.246	0.498
KD‐specific scale												
Sx	87.0 ± 13.3	83.0 ± 15.4	0.123	86.7 ± 13.0	86.3 ± 14.0*	0.849	85.2 ± 18.4	87.4 ± 13.9*	0.441	0.196	0.762	0.008
KD effects	86.1 ± 15.6	84.3 ± 15.7	0.535	85.1 ± 15.2	85.9 ± 16.3	0.801	82.5 ± 20.4	86.8 ± 12.6	0.162	0.719	0.677	0.025
KD burden	65.1 ± 24.4	61.6 ± 23.3	0.410	63.3 ± 26.9	60.2 ± 25.7	0.521	64.6 ± 24.2	59.5 ± 24.1	0.240	0.669	0.321	0.837
Work status	58.6 ± 43.1	51.0 ± 41.4	0.345	53.4 ± 43.8	51.9 ± 43.1	0.855	50.0 ± 41.9	47.1 ± 42.5	0.721	0.081	0.593	0.529
Cognitive function	85.7 ± 17.4	85.7 ± 15.6	0.984	88.3 ± 14.5	84.9 ± 16.9	0.228	85.3 ± 19.0	89.4 ± 13.8*	0.175	0.415	0.936	0.019
QSI	76.7 ± 18.0	76.3 ± 18.1	0.893	78.2 ± 17.1	76.6 ± 18.5	0.629	69.6 ± 15.7*	74.8 ± 13.1	0.049	0.014	0.626	0.156
Sexual function	91.3 ± 16.4	84.2 ± 23.8	0.370	88.5 ± 17.3	85.0 ± 21.2	0.643	78.8 ± 29.5	80.0 ± 24.9	0.911	0.137	0.669	0.691
Sleep	69.7 ± 17.6	67.4 ± 17.3	0.466	66.3 ± 19.0*	67.3 ± 16.3	0.756	67.5 ± 18.0	68.3 ± 17.4	0.815	0.417	0.946	0.403
Social support	69.4 ± 18.8	67.7 ± 23.2	0.646	70.0 ± 20.0	71.3 ± 20.4	0.717	67.5 ± 20.7	69.1 ± 22.1	0.925	0.528	0.932	0.719
OHR	61.8 ± 17.5	58.7 ± 18.4	0.325	62.0 ± 17.9	58.0 ± 16.5	0.199	62.3 ± 19.6	59.8 ± 15.0	0.430	0.737	0.225	0.872

Data are expressed as means ± standard deviations. Comparisons between CON and REN groups were tested using Student's *t* test.

Abbreviations: BP, bodily pain; GH, general health; KD, kidney disease; MCS, mental component scale; MH, mental health; OHR, overall health rating; PCS, physical component scale; PF, physical functioning; QSI, quality of social interaction; RE, role limitations due to emotional problems; RP, role limitations due to physical health problems; SF, social functioning; Sx, symptoms/problems; VT, vitality.

* *P* < 0.05 vs. baseline value.

#### Laboratory findings

There were no significant differences in the serum albumin, hs‐CRP, myostatin, TNF‐α, IL‐6, or eGFR levels; metabolic equivalent of task (MET); or the spot urine protein‐to‐creatinine ratio at baseline, 24 weeks, or 48 weeks between the two groups (*Table* S3). The serum albumin level in the REN group was significantly decreased over 48 weeks, but the magnitude of change was small. The eGFR decreased over 48 weeks in both groups, but difference between the two groups was not significant. MET in the REN group decreased over 48 weeks, but it was not statistically significant in the PP analysis.

## Discussion

We evaluated the effect of AST‐120 on muscle health and QoL in CKD patients and measured gait speed, muscle mass, strength, and QoL over 1 year. IS was decreased in both treatment groups, but the REN group showed a steadier decline. A difference of gait speed ≥0.1 m/s between the two groups was not observed during the study period. HGS decreased within the first 24 weeks and did not significantly change within the next 24 weeks in either treatment group or in the men. Although the proportions of patients with low muscle mass or sarcopenia were higher in the REN group than in the CON group at baseline, the difference between the two treatment groups gradually attenuated throughout the study period. Bodily pain, vitality, symptoms/problems, and cognitive function improved in the REN group, while the quality of social interactions and kidney‐disease effects significantly decreased in the CON group. Interaction between time and group was evident in symptoms/problems, cognitive function, and kidney disease effects.

Recent studies have shown the association between IS and muscle in CKD. The IS level in muscles increased as the serum IS level increased in a CKD mouse model.^21^ Haemodialysis patients with high IS levels exhibited greater decreases in muscle mass and strength than do those with low IS levels.^25^ These results revealed that a decrease in IS levels using the proper intervention may attenuate the development of sarcopenia, but to the best of our knowledge, no studies on the effect of AST‐120 on sarcopenia in nondialysis CKD patients have been conducted.

In this study, there was no significant difference in gait speed between the treatment groups at any of the time points during the study period. We can consider several explanations about the negative result of gait speed. First, small sample size can be a matter. The SD of gait speed was about 0.3 m/s in this study, which was different from that of sample size estimation. The appropriate sample size based on the SD of 0.3 m/s was 175 per treatment group. Second, we cannot rule out the possibility of interoperator variability in measuring gait speed, although we repeatedly educated the assessors on how to measure gait speed in advance. Measurement by using automatic walkway systems can be a solution. Third, the gait speed of participants in this study was lower than that of general population with similar age,^26^ despite of no significant decrease of SMI. This may be related to the effect of uraemic toxin that makes muscle quality worse, including increased intramuscular lipid content,^27^ in addition to ageing. These changes may limit the exercise capacity improvement according to the IS lowering with AST‐120. Fourth, the decrease of IS (30–40% in the REN group throughout the study period) might not be enough to significantly reverse the effect of IS on the gait speed. The normal level of serum IS is around a fourth of that of CKD Stage 3–4,^28^ which is much lower than the final IS level in this study. Other methods to decrease uraemic toxins combined with AST‐120, such as prebiotics or synbiotics,^29^ may result in better outcome in terms of the gait speed.

However, gait speed significantly increased over 1 year only in the REN group. The IS‐induced decline in exercise capacity (running time) associated with decreased mitochondrial‐rich type 1 fibres was significantly restored by AST‐120 treatment in a CKD mouse model.^23^ Our results are consistent with this finding.

Hand grip strength decreased during the first 24 weeks and did not significantly change during the next 24 weeks in either treatment group or in the men. However, HGS changed very little in the women over the study period. The differences between sexes in the IS, myostatin, and eGFR values may be associated with this phenomenon. The levels of serum IS were higher, and the levels of myostatin and eGFR were lower in the women than in the men throughout the study period. The levels of serum albumin, SCr, and AST‐120 medication compliance and the change of IS during the study period were not different between both sexes. The chronicity and severity of kidney disease, resultant serum IS level, and baseline muscle mass, which is reflected by the myostatin level,^30^ can influence the efficacy of AST‐120 in improving muscle strength.

In this study, IS showed a negative correlation with SMI and HGS at each measurement, but the correlation was statistically significant in only the REN group (data not shown).

Aberrations in the IS‐induced pathway to sarcopenia (intracellular IS uptake through OATs, ROS generation, and regulation of TNF‐α, IL‐6, myostatin, and atrogin‐1) could have caused the null effect of AST‐120 on SMI and HGS and could explain the decrease in the proportion of patients with low muscle mass and sarcopenia in the REN group in this study. First, decreased OAT expression due to uraemic conditions^31^ in addition to the competition between uraemic toxins and medications could have oversaturated OATs. By relieving the supersaturated state of OATs through uraemic toxin lowering with AST‐120, the usual muscle reaction of SMI and HGS in response to IS could have been restored. This idea can explain the negative correlation of IS with SMI and HGS in only the REN group and the decreasing proportion of patients with low muscle mass and sarcopenia with the introduction of the AST‐120 in the REN group in this study. Second, the difference in the levels of uraemic toxins other than IS, for example, p‐cresyl sulfate, and metabolic acidosis due to differences in diet can alter the effect of AST‐120. The amount or type of protein intake can influence the uraemic toxin level. The urinary excretion of IS and p‐cresyl sulfate has been found to be significantly lower in vegetarians than in omnivores.^32^ Plant proteins had beneficial effect on the acid load more than animal protein.^33^, ^34^ Metabolic acidosis can increase sensitivity to ROS^35^ and induce glucocorticoid secretion and insulin resistance, which is associated with decreased muscle protein synthesis.^36^, ^37^, ^38^ Unfortunately, we did not evaluate the diet of the participants. Third, other pathway to sarcopenia may have been activated, such as atrogin‐1 pathway.^13^ However, we did not measure the level of atrogin‐1. Nevertheless, the negative correlation of IS with SMI and GHS in only the REN group needs to be studied further.

A meaningful difference or trend was not observed in muscle mass in either group. A decrease in muscle strength or physical performance can develop earlier than a decrease in muscle mass, which is mainly caused by mitochondrial dysfunction in skeletal muscles.^7^ One year is a relatively short period to detect the effect of IS and AST‐120 on muscle mass and studies with longer durations are needed. However, the proportions of participants with low muscle mass or sarcopenia showed a decreasing tendency in the REN group. This finding may be related to the muscle mass change caused by the AST‐120 or IS levels of the participants with values near the cutoff value.

The other important result of our study is the effect of AST‐120 on QoL. In the REN group, bodily pain improved according to the ITT and PP analyses, and vitality improved according to the PP analysis, which could be associated with improvements in overall muscle health. Symptoms/problems also improved in the REN group. IS as a uraemic toxin can be associated with uraemic symptoms in various organs, and the use of AST‐120 may have improved subjective symptoms/problems via a decrease in IS. Cognitive function improved in the REN group alone. Yeh *et al*. showed a positive association between the IS level and cognitive impairment, and our results are consistent with this result.^39^

Our study has inherent limitations. First, the expected gait speed change over the study period was not observed. Because the gait speed of the CON group showed a stationary or decreasing tendency and the of the REN group showed a significant increase over 1 year, we may need to conduct a study over a longer period of time to detect a gait speed difference between the two groups. Second, we did not appropriately evaluate life style and physical activity, which can be confounding factors of muscle health. Physical activity was only evaluated by using the IPAQ, but its limitation is that it involves subjective recall. Third, our study was not placebo‐controlled and blinded. It can particularly influence on the subjective outcomes such as QoL scores and physical activities. We tried to perform this randomized study with the least biases. However, non‐blindness can make performance biases, which can be a significant confounding factor of outcome measurements.

In conclusion, the addition of AST‐120 to standard treatment did not make a significant difference in gait speed. Prospective studies with longer follow‐up duration, larger sample size, interventions of uraemic toxin lowering added to AST‐120, and automatic measurement systems are necessary to elucidate the role of AST‐120 and uraemic toxin in sarcopenia in CKD patients.

## Conflict of interest

The authors have no conflicts of interest to disclose.

## Ethical statement

The study was performed in accordance with the Declaration of Helsinki. All participants provided written informed consent. The manuscript complies with the ethical guidelines for authorship and publishing in the *Journal of Cachexia, Sarcopenia and Muscle*.

## Supporting information


**Figure S1.** Changes of standing handgrip strength in men (a) and women (b) from baseline to 24 and 48 weeks. Data were expressed as mean and standard error. *p < 0.05 vs. baseline, ^¶^ *p* < 0.01 vs. baseline. ITT, intention‐to‐treat; PP, per‐ protocol.

**Table S1.** The effect of AST‐120 on the progression of static‐start gait speed during the study period.
**Table S2.** Comparison of quality of life scale scores according to group in per‐protocol population.
**Table S3.** Comparison of biochemistry between treatment groups.

## References

[1] Moon SJ , Kim TH , Yoon SY , Chung JH , Hwang HJ . Relationship between stage of chronic kidney disease and sarcopenia in Korean aged 40 years and older using the Korea National Health and Nutrition Examination Surveys (KNHANES IV‐2, 3, and V‐1, 2), 2008–2011. PLoS One 2015;10:e0130740. 26083479 10.1371/journal.pone.0130740 PMC4470593

[2] Foley RN , Wang C , Ishani A , Collins AJ , Murray AM . Kidney function and sarcopenia in the United States general population: NHANES III. Am J Nephrol 2007;27:279–286. 17440263 10.1159/000101827

[3] Lenk K , Schuler G , Adams V . Skeletal muscle wasting in cachexia and sarcopenia: molecular pathophysiology and impact of exercise training. J Cachexia Sarcopenia Muscle 2010;1:9–21. 21475693 10.1007/s13539-010-0007-1 PMC3060644

[4] Wang XH , Mitch WE . Mechanisms of muscle wasting in chronic kidney disease. Nat Rev Nephrol 2014;10:504–516. 24981816 10.1038/nrneph.2014.112 PMC4269363

[5] Adey D , Kumar R , McCarthy JT , Nair KS . Reduced synthesis of muscle proteins in chronic renal failure. Am J Physiol Endocrinol Metab 2000;278:E219–E225. 10662705 10.1152/ajpendo.2000.278.2.E219

[6] Yazdi PG , Moradi H , Yang JY , Wang PH , Vaziri ND . Skeletal muscle mitochondrial depletion and dysfunction in chronic kidney disease. Int J Clin Exp Med 2013;6:532–539. 23936591 PMC3731184

[7] Tamaki M , Miyashita K , Wakino S , Mitsuishi M , Hayashi K , Itoh H . Chronic kidney disease reduces muscle mitochondria and exercise endurance and its exacerbation by dietary protein through inactivation of pyruvate dehydrogenase. Kidney Int 2014;85:1330–1339. 24284514 10.1038/ki.2013.473

[8] Deguchi T , Kouno Y , Terasaki T , Takadate A , Otagiri M . Differential contributions of rOat1 (Slc22a6) and rOat3 (Slc22a8) to the in vivo renal uptake of uremic toxins in rats. Pharm Res 2005;22:619–627. 15846470 10.1007/s11095-005-2486-x

[9] Miyamoto Y , Watanabe H , Noguchi T , Kotani S , Nakajima M , Kadowaki D , et al. Organic anion transporters play an important role in the uptake of p‐cresyl sulfate, a uremic toxin, in the kidney. Nephrol Dial Transplant 2011;26:2498–2502. 21303967 10.1093/ndt/gfq785

[10] Deguchi T , Kusuhara H , Takadate A , Endou H , Otagiri M , Sugiyama Y . Characterization of uremic toxin transport by organic anion transporters in the kidney. Kidney Int 2004;65:162–174. 14675047 10.1111/j.1523-1755.2004.00354.x

[11] Deguchi T , Ohtsuki S , Otagiri M , Takanaga H , Asaba H , Mori S , et al. Major role of organic anion transporter 3 in the transport of indoxyl sulfate in the kidney. Kidney Int 2002;61:1760–1768. 11967025 10.1046/j.1523-1755.2002.00318.x

[12] Motojima M , Hosokawa A , Yamato H , Muraki T , Yoshioka T . Uremic toxins of organic anions up‐regulate PAI‐1 expression by induction of NF‐kappaB and free radical in proximal tubular cells. Kidney Int 2003;63:1671–1680. 12675842 10.1046/j.1523-1755.2003.00906.x

[13] Enoki Y , Watanabe H , Arake R , Sugimoto R , Imafuku T , Tominaga Y , et al. Indoxyl sulfate potentiates skeletal muscle atrophy by inducing the oxidative stress‐mediated expression of myostatin and atrogin‐1. Sci Rep 2016;6:32084. 27549031 10.1038/srep32084 PMC4994088

[14] Niwa T . Role of indoxyl sulfate in the progression of chronic kidney disease and cardiovascular disease: experimental and clinical effects of oral sorbent AST‐120. Ther Apher Dial 2011;15:120–124. 21426500 10.1111/j.1744-9987.2010.00882.x

[15] Watanabe H , Miyamoto Y , Honda D , Tanaka H , Wu Q , Endo M , et al. p‐Cresyl sulfate causes renal tubular cell damage by inducing oxidative stress by activation of NADPH oxidase. Kidney Int 2013;83:582–592. 23325087 10.1038/ki.2012.448

[16] Sato E , Mori T , Mishima E , Suzuki A , Sugawara S , Kurasawa N , et al. Metabolic alterations by indoxyl sulfate in skeletal muscle induce uremic sarcopenia in chronic kidney disease. Sci Rep 2016;6:36618. 27830716 10.1038/srep36618 PMC5103201

[17] Shimizu H , Bolati D , Adijiang A , Adelibieke Y , Muteliefu G , Enomoto A , et al. Indoxyl sulfate downregulates renal expression of Klotho through production of ROS and activation of nuclear factor‐kB. Am J Nephrol 2011;33:319–324. 21389697 10.1159/000324885

[18] Adijiang A , Shimizu H , Higuchi Y , Nishijima F , Niwa T . Indoxyl sulfate reduces klotho expression and promotes senescence in the kidneys of hypertensive rats. J Ren Nutr 2011;21:105–109. 21195930 10.1053/j.jrn.2010.10.020

[19] Avin KG , Coen PM , Huang W , Stolz DB , Sowa GA , Dubé JJ , et al. Skeletal muscle as a regulator of the longevity protein, Klotho. Front Physiol 2014;5:10.3389/fphys.2014.00189 PMC4060456 24987372

[20] Semba RD , Ferrucci L , Sun K , Simonsick E , Turner R , Milijkovic I , et al. Low plasma Klotho concentrations and decline of knee strength in older aAdults. J Gerontol A Biol Sci Med Sci 2016;71:103–108. 26359247 10.1093/gerona/glv077 PMC4706099

[21] Sato E , Saigusa D , Mishima E , Uchida T , Miura D , Morikawa‐Ichinose T , et al. Impact of the oral adsorbent AST‐120 on organ‐specific accumulation of uremic toxins: LC–MS/MS and MS imaging techniques. Toxins (Basel) 2017;10:10.3390/toxins10010019 PMC5793106 29283413

[22] Adijiang A , Niwa T . An oral sorbent, AST‐120, increases Klotho expression and inhibits cell senescence in the kidney of uremic rats. Am J Nephrol 2010;31:160–164. 19955715 10.1159/000264634

[23] Enoki Y , Watanabbe H , Arake R , Fujimura R , Ishiodori K , Imafuku T , et al. Potential therapeutic interventions for chronic kidney disease‐associated sarcopenia via indoxyl sulfate‐induced mitochondrial dysfunction. J Cachexia Sarcopenia Muscle 2017;8:735–747. 28608457 10.1002/jcsm.12202 PMC5659061

[24] Peel NM , Kuys SS , Klein K . Gait speed as a measure in geriatric assessment in clinical settings: a systematic review. J Gerontol A Biol Sci Med Sci 2013;68:39–46. 22923430 10.1093/gerona/gls174

[25] Lin YL , Liu CH , Lai YH , Wang CH , Kuo CH , Liou HH , et al. Association of Serum Indoxyl Sulfate Levels with Skeletal Muscle Mass and Strength in Chronic Hemodialysis Patients: A 2‐year Longitudinal Analysis. Calcif Tissue Int 2020;107:257–265. 32691117 10.1007/s00223-020-00719-x

[26] Lau LK , Wee SL , Pang WJB , Chen KK , Abdul Jabbar K , Yap PLK , et al. Reference values of gait sped and gait spatiotemporal parameters for a South East Asian population: The Yishun Study. Clin Interv Aging 2020;15:1753–1765. 33061327 10.2147/CIA.S270407 PMC7522423

[27] Lustgarten MS . The kidney‐gut‐muscle axis in end‐stage renal disease is similarly represented in older adults. Nutrients 2020;12:10.3390/nu12010106 PMC7019845 31905970

[28] Lin CN , Wu IW , Huang YF , Peng SY , Huang YC , Ning HC . Measuring serum total and free indoxyl sulfate and p‐cresyl sulfate in chronic kidney disease using UPLC‐MS/MS. J Food Drug Anal 2019;27:502–509. 30987721 10.1016/j.jfda.2018.10.008 PMC9296214

[29] Takkavatakarn K , Wuttiputinun T , Phannajit J , Praditpornsilpa K , Eiam‐Ong S , Susantitaphong P . Protein‐bound uremic toxin lowering strategies in chronic kidney disease: a systematic review and meta‐analysis. J Nephrol 2021; 10.1007/s40620-020-00955-2 33484425

[30] Baczek J , Silkiewicz M , Wojszel ZB . Myostatin as a biopmarker of muscle wasting and other pathologies‐State of the art and knowledge gaps. Nutrients 2020; 10.3390/nu12082401 PMC7469036 32796600

[31] Naud J , Michaud J , Beauchemin S , Hébert MJ , Roger M , Lefrancois S , et al. Effects of chronic renal failure on kidney drug transporters and cytochrome P450 in rats. Drug Metab Dispos 2011;39:1363–1369. 21525170 10.1124/dmd.111.039115

[32] Patel KP , Luo FH , Plummer NS , Hostetter TH , Meyer TW . The production of p‐cresol sulfate and indoxyl sulfate in vegetarians versus omnivores. Clin J Am Soc Nephrol 2012;7:982–988. 22490877 10.2215/CJN.12491211 PMC3362314

[33] Frassetto LA , Todd KM , Morris RC Jr , Sebastian A . Estimation of net endogenous noncarbonic acid production in humans from diet potassium and protein contents. Am J Clin Nutr 1998;68:576–583. 9734733 10.1093/ajcn/68.3.576

[34] Remer T , Manz F . Potential renal acid load of foods and its influence on urine pH. J Am Diet Assoc 1995;95:791–797. 7797810 10.1016/S0002-8223(95)00219-7

[35] Lamonte G , Tang X , Chen JL , Wu J , Ding CK , Keenan MM , et al. Acidosis induces reprogramming of cellular metabolism to mitigate oxidative stress. Cancer Metab 2013;1:10.1186/2049-3002-1-23 PMC4178214 24359630

[36] Kalantar‐Zadeh K , Mehrotra R , Fouque D , Kopple JD . Metabolic acidosis and malnutrition‐inflammation complex syndrome. Semin Dial 2004;17:455–465. 15660576 10.1111/j.0894-0959.2004.17606.x

[37] Kalantar‐Zadeh K , Fouque D . Nutritional management of chronic kidney disease. N Engl J Med 2017;377:1765–1776. 29091561 10.1056/NEJMra1700312

[38] Bailey JL , Zheng B , Hu Z , Price SR , Mitch WE . Chronic kidney disease causes defects in signaling through the insulin receptor substrate/phosphatidylinositol‐e‐kinase/Akt pathway: implications for muscle atrophy. J Am Soc Nephrol 2006;17:1388–1394. 16611720 10.1681/ASN.2004100842

[39] Yeh YC , Huang MF , Liang SS , Hwang SJ , Tsai JC , Liu TL , et al. Indoxyl sulfate, not p‐cresyl sulfate, is associated with cognitive impairment in early‐stage chronic kidney disease. Neurotoxicology 2016;53:148–152. 26797588 10.1016/j.neuro.2016.01.006

