# Supplementary material for: Effects of AST‐120 on muscle health and quality of life in chronic kidney disease patients: results of RECOVERY study
Source: J Cachexia Sarcopenia Muscle. 2021 Dec 3;13(1):397–408. doi: 10.1002/jcsm.12874 (PMC8818653; doi:10.1002/jcsm.12874)
Supplement: Supplementary file 1 — Figure S1. Changes of standing handgrip strength in men (a) and women (b) from baseline to 24 and 48 weeks. Data were expressed as mean and standard error. *p < 0.05 vs. baseline, ¶ p < 0.01 vs. baseline. ITT, intention‐to‐treat; PP, per‐ protocol. [file JCSM-13-397-s002.ppt]

## Slide 1
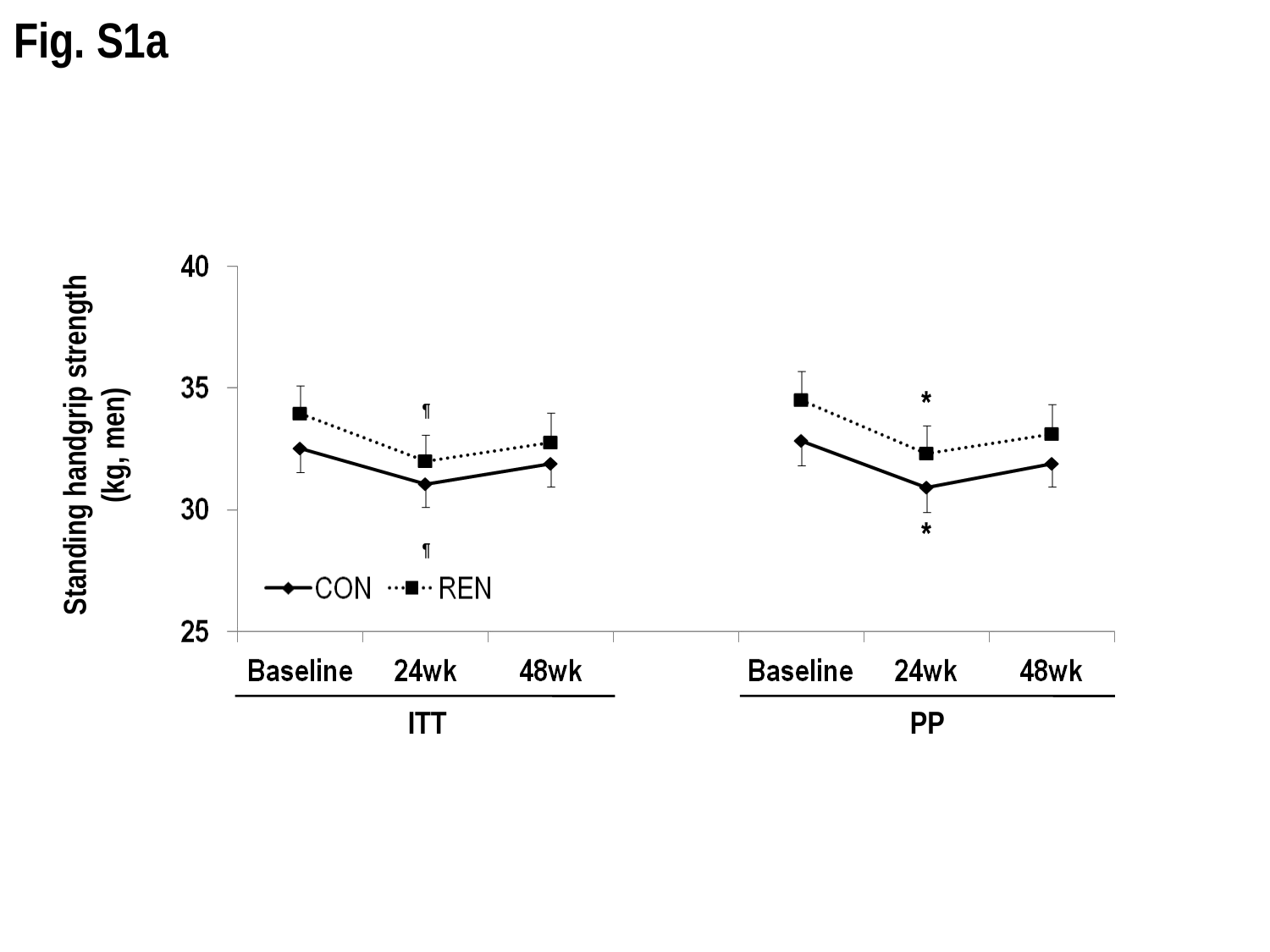

Fig. S1a
*
¶
Standing handgrip strength (kg, men)
*
¶
ITT
PP

## Slide 2
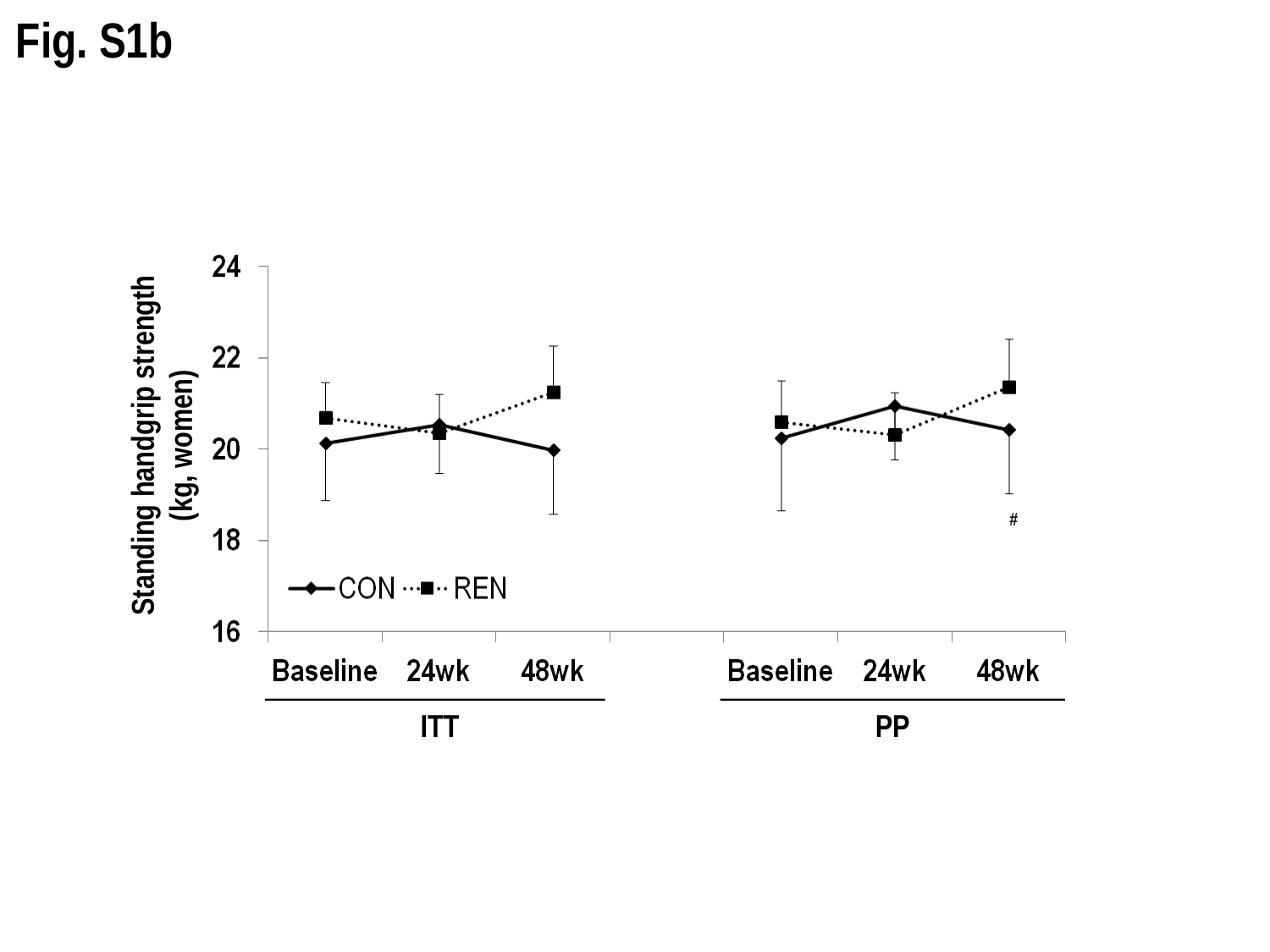

Fig. S1b
Standing handgrip strength (kg, women)
#
ITT
PP
