# Supplementary material for: Effects of AST‐120 on muscle health and quality of life in chronic kidney disease patients: results of RECOVERY study
Source: J Cachexia Sarcopenia Muscle. 2021 Dec 3;13(1):397–408. doi: 10.1002/jcsm.12874 (PMC8818653; doi:10.1002/jcsm.12874)
Supplement: Supplementary file 2 — Table S1. The effect of AST‐120 on the progression of static‐start gait speed during the study period. Table S2. Comparison of quality of life scale scores according to group in per‐protocol population. Table S3. Comparison of biochemistry between treatment groups. [file JCSM-13-397-s001.doc]

**Table S1. The effect of AST-120 on the progression of static-start** gait speed during the study period

|  | Intention-to-treat population | | | Per-protocol population | | |
| --- | --- | --- | --- | --- | --- | --- |
|  | Standardized coefficient | 95% confidence interval | *p*-value | Standardized coefficient | 95% confidence interval | *p* -value |
| Age (year) | -0.138 | -0.005–0.001 | 0.13 | -0.152 | -0.005–0.000 | 0.09 |
| Sex (female) | -0.010 | -0.072–0.064 | 0.91 | -0.009 | -0.073–0.066 | 0.92 |
| Randomization (REN) | 0.176 | 0.000–0.128 | 0.05 | 0.17 | -0.003–0.128 | 0.06 |
| Primary disease (DM) | 0.108 | -0.026–0.106 | 0.23 | 0.119 | -0.023–0.111 | 0.19 |
| Physical activity  (IPAQ-MET total) | 0.137 | 0.000–0.000 | 0.12 | 0.135 | 0.000–0.000 | 0.13 |

DM, diabetes mellitus; IPAQ, international physical activity questionnaire; MET, metabolic equivalent of task; REN, renamezin®

**Table S2. Comparison of quality of life scale scores according to group in per-protocol population**

|  |  | Baseline | | |  | | | | 6M | |  | | 12 M | | |  | | | *p* for interaction | | | | | | |
| --- | --- | --- | --- | --- | --- | --- | --- | --- | --- | --- | --- | --- | --- | --- | --- | --- | --- | --- | --- | --- | --- | --- | --- | --- | --- |
| Short form-6 scale |  | CON | REN | *p* | |  | CON | REN | | *p* |  | CON | | REN | *p* | |  | Time | | | Group | | Time-Group | | |
| PF |  | 80.2 ± 18.9 | 76.4 ± 22.7 | 0.33 | |  | 78.7 ± 20.4 | 78.5 ± 20.1 | | 0.96 |  | 78.1 ± 25.2 | | 80.3 ± 18.3 | 0.58 | |  | | | 0.79 | | 0.87 | 0.14 |  | |
| RP |  | 75.0 ± 33.6 | 69.2 ± 39.2 | 0.57 | |  | 77.3 ± 36.1 | 66.3 ± 42.1 | | 0.11 |  | 70.7 ± 41.9 | | 69.2 ± 40.0 | 0.83 | |  | | | 0.78 | | 0.30 | 0.29 |  | |
| BP |  | 77.7 ± 22.6 | 75.7 ± 24.3 | 0.64 | |  | 81.4 ± 23.3 | 77.7 ± 24.7 | | 0.39 |  | 78.7 ± 20.2 | | 83.4 ± 20.2* | 0.25 | |  | | 0.10 | | | 0.93 | 0.14 |  | |
| GH |  | 44.2 ± 17.1 | 43.8 ± 16.0 | 0.87 | |  | 45.5 ± 17.0 | 47.1 ± 18.5 | | 0.63 |  | 44.1 ± 16.5 | | 45.6 ± 18.2 | 0.64 | |  | | 0.18 | | | 0.76 | 0.67 |  | |
| VT |  | 51.5 ± 18.4 | 48.5 ± 17.7 | 0.35 | |  | 49.8 ± 16.1 | 51.3 ± 18.8 | | 0.61 |  | 51.6 ± 16.3 | | 52.3 ± 15.6* | 0.78 | |  | | 0.36 | | | 0.94 | 0.21 |  | |
| SF |  | 87.7 ± 21.6 | 82.5 ± 23.4 | 0.20 | |  | 84.4 ± 21.2 | 82.3 ± 22.6 | | 0.59 |  | 82.4 ± 23.3 | | 83.5 ± 22.8 | 0.78 | |  | | 0.52 | | | 0.55 | 0.30 |  | |
| RE |  | 77.1 ± 38.9 | 68.9 ± 42.5 | 0.26 | |  | 74.0 ± 41.3 | 70.6 ± 42.6 | | 0.65 |  | 70.8 ± 42.2 | | 65.0 ± 44.9 | 0.45 | |  | | 0.27 | | | 0.37 | 0.77 |  | |
| MH |  | 65.2 ± 15.4 | 65.9 ± 15.4 | 0.23 | |  | 65.8 ± 14.4 | 61.7 ± 15.2 | | 0.13 |  | 65.1 ± 14.2 | | 61.2 ± 15.1 | 0.13 | |  | | 0.91 | | | 0.09 | 0.96 |  | |
| OHR |  | 27.0 ± 16.8 | 25.4 ± 16.9 | 0.61 | |  | 31.6 ± 20.0* | 34.2 ± 19.5* | | 0.47 |  | 33.2 ± 20.9* | | 34.2 ± 20.6* | 0.79 | |  | | <0.01 | | | 0.82 | 0.46 |  | |
| PCS |  | 69.3 ± 18.4 | 66.3 ± 20.4 | 0.39 | |  | 70.7 ± 19.9 | 67.4 ± 20.5 | | 0.35 |  | 67.9 ± 22.9 | | 69.6 ± 18.6 | 0.64 | |  | | 0.64 | | | 0.63 | 0.16 |  | |
| MCS |  | 70.4 ± 18.4 | 65.4 ± 19.9 | 0.15 | |  | 68.5 ± 18.6 | 66.5 ± 20.6 | | 0.57 |  | 67.5 ± 18.9 | | 65.5 ± 19.9 | 0.57 | |  | | 0.64 | | | 0.34 | 0.46 |  | |
| KD-specific scale |  |  |  |  | |  |  |  | |  |  |  | |  |  | |  | |  | | |  |  | |  |
| Sx |  | 87.0 ± 13.4 | 83.0 ± 15.6 | 0.13 | |  | 86.7 ± 13.1 | 86.2 ± 14.1* | | 0.84 |  | 85.1 ± 18.5 | | 87.4 ± 14.0* | 0.42 | |  | | 0.23 | | | 0.78 | 0.01 | |  |
| KD effects |  | 86.1 ± 15.7 | 84.4 ± 15.8 | 0.54 | |  | 85.1 ± 15.3 | 85.8 ± 16.4 | | 0.80 |  | 82.4 ± 20.5* | | 86.6 ± 12.6 | 0.17 | |  | | 0.67 | | | 0.68 | 0.03 | |  |
| KD burden |  | 65.5 ± 24.3 | 62.0 ± 23.3 | 0.40 | |  | 63.3 ± 27.1 | 60.7 ± 25.7 | | 0.59 |  | 64.5 ± 24.4 | | 59.8 ± 24.2 | 0.28 | |  | | 0.59 | | | 0.36 | 0.84 | |  |
| Work status |  | 58.6 ± 43.1 | 52.0 ± 41.2 | 0.41 | |  | 50.0 ± 44.5 | 46.7 ± 43.0 | | 0.67 |  | 40.9 ± 42.6 | | 40.8 ± 42.7 | 0.43 | |  | | 0.07 | | | 0.69 | 0.51 | |  |
| Cognitive function |  | 85.7 ± 17.6 | 85.4 ± 15.6 | 0.92 | |  | 88.1 ± 14.6 | 85.1 ± 16.9 | | 0.28 |  | 85.1 ± 19.1 | | 89.2 ± 13.9* | 0.17 | |  | | 0.43 | | | 0.92 | 0.03 | |  |
| QSI |  | 76.6 ± 18.1 | 76.4 ± 18.2 | 0.97 | |  | 78.0 ± 17.2 | 76.6 ± 18.7 | | 0.65 |  | 69.7 ± 15.8* | | 74.6 ± 13.1 | 0.06 | |  | | 0.02 | | | 0.62 | 0.20 | |  |
| Sexual function |  | 83.6 ± 24.0 | 81.5 ± 21.9 | 0.78 | |  | 81.5 ± 23.9 | 83.1 ± 21.2 | | 0.82 |  | 73.9 ± 31.8 | | 82.4 ± 24.2 | 0.36 | |  | | 0.14 | | | 0.67 | 0.69 | |  |
| Sleep |  | 69.5 ± 17.7 | 68.1 ± 16.5 | 0.63 | |  | 65.9 ± 18.8 | 67.7 ± 16.1 | | 0.55 |  | 67.0 ± 17.7 | | 67.9 ± 17.3 | 0.77 | |  | | 0.30 | | | 0.88 | 0.43 | |  |
| Social support |  | 69.5 ± 18.9 | 69.5 ± 18.9 | 0.75 | |  | 70.0 ± 20.2 | 71.4 ± 20.6 | | 0.71 |  | 69.5 ± 20.9 | | 69.2 ± 22.3 | 0.92 | |  | | 0.60 | | | 0.98 | 0.79 | |  |
| OHR |  | 61.6 ± 17.5 | 58.7 ± 18.5 | 0.37 | |  | 61.9 ± 18.0 | 58.5 ± 16.2 | | 0.27 |  | 62.3 ± 19.7 | | 59.8 ± 15.1 | 0.43 | |  | | 0.75 | | | 0.27 | 0.96 | |  |

Data are expressed as means ± standard deviations. Comparisons between CON and REN groups were tested using Student’s *t*-test.

Abbreviations: BP, bodily pain; CON, control; GH, general health; KD, kidney disease; MCS, mental component scale; MH, mental health; OHR, overall health rating; PCS, physical component scale; PF, physical functioning; QSI, quality of social interaction; RE, role limitations due to emotional problems; REN, renamezin®; RP, role limitations due to physical health problems; SF, social functioning; Sx, symptoms/problems; VT, vitality. **p* < 0.05 vs. baseline value

**Table S3. Comparison of biochemistry between treatment groups**

|  | Baseline | | | 24 weeks | | | 48 weeks | | |
| --- | --- | --- | --- | --- | --- | --- | --- | --- | --- |
|  | CON | REN | *p*-value | CON | REN | *p*-value | CON | REN | *p*-value |
| ITT population |  |  |  |  |  |  |  |  |  |
| Albumin (g/dL) | 4.3 ± 0.4 | 4.3 ± 0.3 | 0.24 | 4.2 ± 0.4 | 4.3 ± 0.4* | 0.45 | 4.2 ± 0.4 | 4.2 ± 0.4* | 0.95 |
| hs-CRP (mg/dL) | 0.4 ± 1.0 | 0.5 ± 1.4 | 0.59 | 0.3 ± 0.5 | 0.4 ± 0.9 | 0.43 | 0.5 ± 2.1 | 0.4 ± 1.1 | 0.78 |
| Myostatin (pg/mL) | 4.7 ± 2.0 | 4.9 ± 2.1 | 0.72 | 4.3 ± 2.1 | 4.4 ± 2.6 | 0.78 | 3.8 ± 1.7 | 4.1 ± 2.0 | 0.40 |
| TNF-α (pg/mL) | 2.0 ± 1.9 | 1.8 ± 1.3 | 0.40 | 1.8 ± 0.7 | 1.9 ± 1.7 | 0.77 | 2.3 ± 5.0 | 1.7 ± 1.1 | 0.34 |
| IL-6 (pg/mL) | 2.2 ± 1.2 | 2.6 ± 2.2 | 0.18 | 4.6 ± 9.7* | 4.1 ± 6.6* | 0.75 | 4.2 ± 10.6* | 5.2 ± 7.8* | 0.53 |
| eGFR | 33.0 ± 12.0 | 34.6 ± 12.9 | 0.44 | 31.0 ± 13.2* | 32.7 ± 14.3* | 0.47 | 32.0 ± 14.2* | 33.1 ± 14.9* | 0.67 |
| MET | 4022 ± 6383 | 3545 ± 7192 | 0.66 | 2099 ± 2067 | 1924 ± 2242 | 0.62 | 2109 ± 3914 | 1738 ± 2324* | 0.48 |
| P/Cr ratio | 1.5 ± 1.7 | 1.2 ± 1.2 | 0.30 | 1.4 ± 1.7 | 1.3 ± 1.6 | 0.65 | 1.5 ± 2.3 | 1.6 ± 1.8 | 0.67 |
| PP population |  |  |  |  |  |  |  |  |  |
| Albumin (g/dL) | 4.3 ± 0.4 | 4.3 ± 0.4 | 0.32 | 4.2 ± 0.3 | 4.2 ± 0.4* | 0.58 | 4.2 ± 0.4 | 4.3 ± 0.4* | 0.90 |
| hs-CRP (mg/dL) | 0.4 ± 1.1 | 0.6 ± 1.5 | 0.54 | 0.3 ± 0.6 | 0.4 ± 0.8 | 0.58 | 0.5 ± 2.1 | 0.4 ± 1.1 | 0.78 |
| Myostatin (pg/mL) | 4.9 ± 2.0 | 4.8 ± 1.7 | 0.88 | 4.3 ± 2.1 | 4.5 ± 2.7 | 0.64 | 3.9 ± 1.7 | 4.1 ± 2.0 | 0.45 |
| TNF-α (pg/mL) | 2.0 ± 1.9 | 1.8 ± 1.4 | 0.51 | 1.8 ± 0.7 | 1.9 ± 1.7 | 0.73 | 2.3 ± 5.0 | 1.7 ± 1.1 | 0.34 |
| IL-6 (pg/mL) | 2.2 ± 1.2 | 2.6 ± 2.4 | 0.25 | 4.7 ± 10.1 | 4.3 ± 6.9* | 0.82 | 4.3 ± 10.8 | 5.4 ± 7.9* | 0.53 |
| eGFR (CKD-EPI) | 33.5 ± 12.0 | 35.1 ± 12.9 | 0.48 | 32.0 ± 13.1* | 33.6 ± 14.1* | 0.50 | 32.2 ± 14.4 | 32.9 ± 15.0* | 0.78 |
| MET | 4199 ± 6704 | 4047 ± 7888 | 0.90 | 2544 ± 2736 | 2330 ± 2598 | 0.65 | 2773 ± 4844 | 2082 ± 2453 | 0.32 |
| Pr/Cr ratio | 1.4 ± 1.7 | 1.3 ± 1.2 | 0.61 | 1.4 ± 1.7 | 1.4 ± 1.6 | 0.96 | 1.5 ± 2.3 | 1.6 ± 1.8 | 0.76 |

Data are expressed as means ± standard deviations. The comparison between groups was tested with *t*-test and comparison with baseline value was tested with paired *t*-test. **p* < 0.05 vs. baseline value

Abbreviations: ITT, intention-to-treat; PP, per-protocol; CON, control; eGFR, estimated glomerular filtration rate; hs-CRP, high sensitivity-C-reactive protein; MET, metabolic equivalent of task; Pr/Cr, spot urine protein to creatinine; REN, renamezin®; TNF-α, tumor necrosis factor-α
